# Supplementary material for: Magnetic droplet soliton pairs
Source: Nat Commun. 2024 Mar 8;15:2118. doi: 10.1038/s41467-024-46404-7 (PMC10923811; doi:10.1038/s41467-024-46404-7)
Supplement: Supplementary file 1 — Supplementary information [file 41467_2024_46404_MOESM1_ESM.pdf]

Supplementary information for Magnetic droplet soliton pairs

S. Jiang[1,2*], S. Chung[2,3,*], M. Ahlberg[2,*], A. Frisk[2], R. Khymyn[2], Q. Tuan Le[2,4], H. Mazraati[4], A. Houshang[2], O. Heinonen[5,†] & J. Åkerman[2,4,6,7]

[1]*School of Microelectronics, South China University of Technology, 511442 Guangzhou, China*

[2]*Physics Department, University of Gothenburg, 412 96, Gothenburg, Sweden*

[3]*Department of Physics Education, Korea National University of Education, Cheongju 28173, Korea*

[4]*Department of Applied Physics, School of Engineering Sciences, KTH Royal Institute of Technology, 100 44 Stockholm, Sweden*

[5]*Materials Science Division, Argonne National Laboratory, Lemont, IL 60439, USA*

[6]*Center for Science and Innovation in Spintronics, Tohoku University, 2-1-1 Katahira, Aoba-ku, Sendai 980-8577 Japan*

[7]*Research Institute of Electrical Communication, Tohoku University, 2-1-1 Katahira, Aoba-ku, Sendai 980-8577 Japan*

[*]*These authors contributed equally to this work.* [†]*Present and permanent address: Seagate Technology, 7801 Computer Ave., Bloomington, MN 55435*

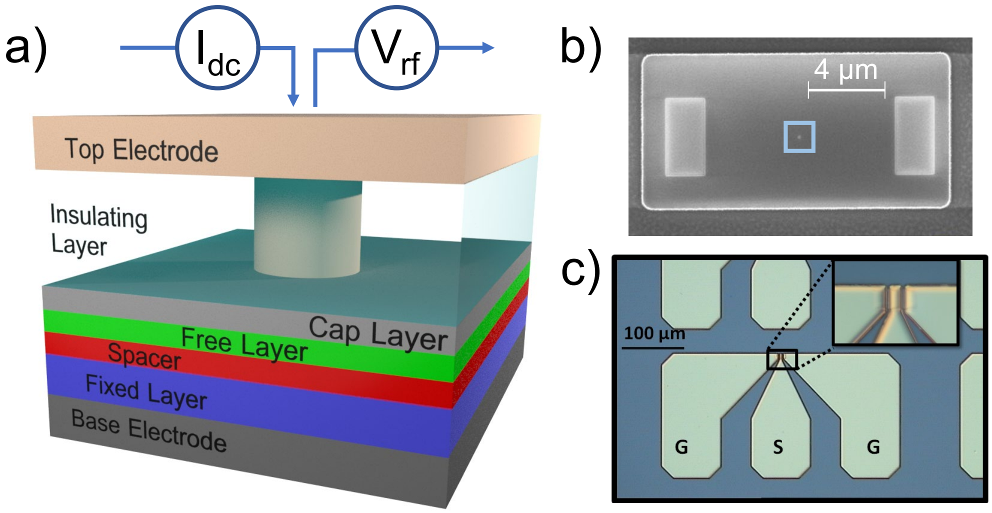

Figure S1: **Supplementary Fig. 1.** **a** Schematic of a nanocontact STNO, zooming in on the nanocontact region. **b** Scanning Electron Microscopy image of a device before the top contact fabrication step. An etched nanohole (highlighted by the blue square) is located in the middle of the mesa, flanked to the left and right by two much larger rectangular openings for the ground contacts. **c** Optical microscopy image of the top gold Ground-Signal-Ground pads on top of the device shown in **b**.

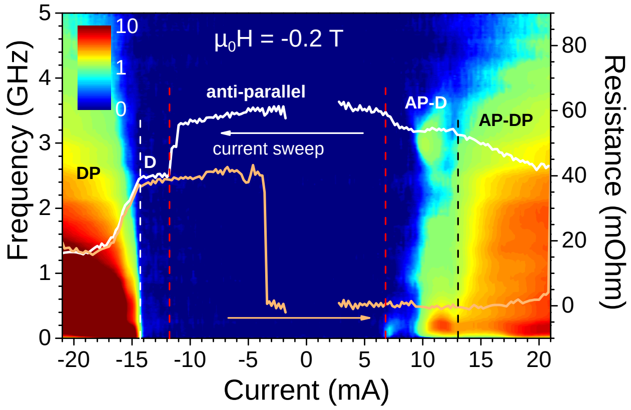

Figure S2: **Supplementary Fig. 2.** Same data as Fig. 2b in the main text, but with the color map on a logarithmic scale. The dc resistance (white line) and corresponding PSD (color map) of a current sweep from $I = 21$ mA to $I = -21$ mA, at $\mu_0 H = -0.2$ T. The orange line shows the resistance of the backward sweep $I = -21 \rightarrow 21$ mA; the corresponding PSD is not shown. AP, D, and DP denote Anti-Parallel, single Droplet, and Droplet Pair, respectively.

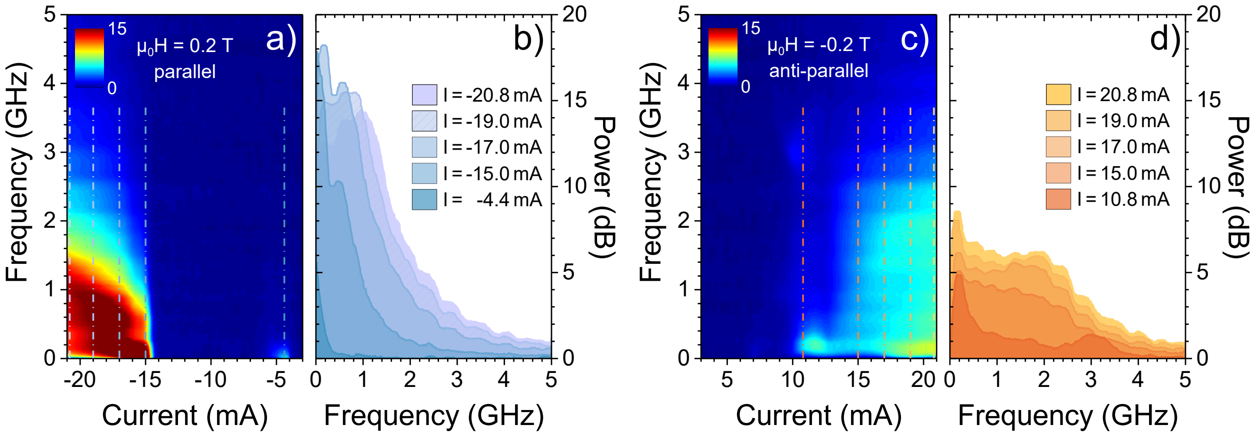

Figure S3: **Supplementary Fig. 3.** Same data as Fig. 2 in the main text, with 2D slices of the color map. The PSD (color map) of a current sweep from $I = 21$ mA to $I = -21$ mA, at **a** $\mu_0 H = +0.2$ T and **c** $\mu_0 H = -0.2$ T. The vertical lines in **a** and **c** correspond to the presented currents in **b** and **d**. The amplitude of the signal is given in dB over noise floor. The small wiggles in the signal is an artifact due to standing waves in one or more of the cables between the microwave components in the measurement chain.

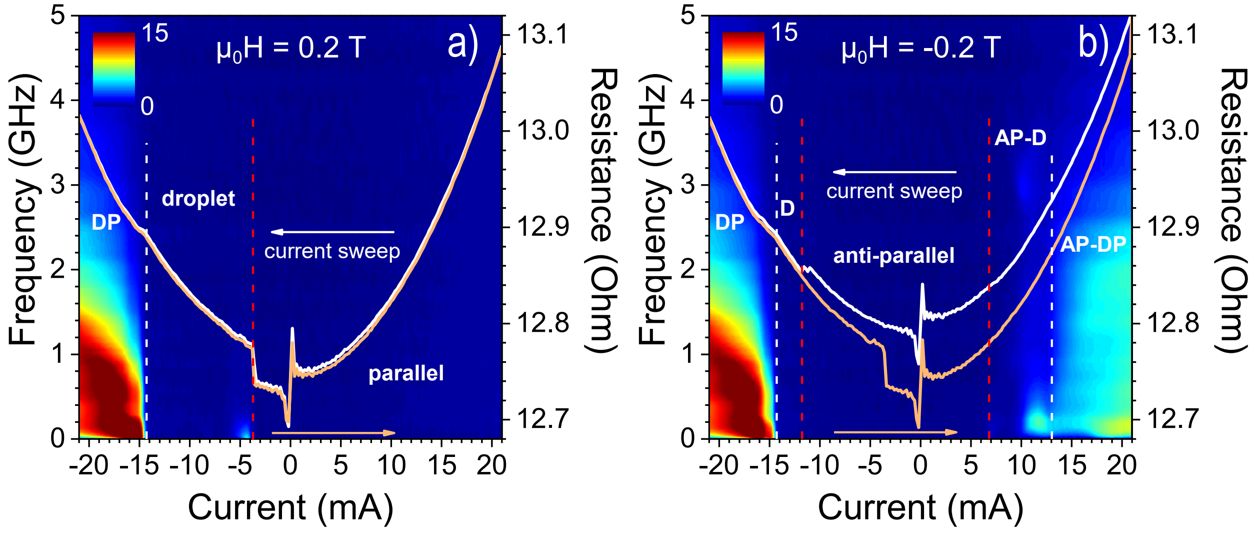

Figure S4: **Supplementary Fig. 4.** Same data as Fig. 2 in the main text, but without background correction of the resistance. The dc resistance (white line) and corresponding PSD (color map) of a current sweep from $I = 21$ mA to $I = -21$ mA, at **a** $\mu_0 H = +0.2$ T and **b** $\mu_0 H = -0.2$ T. The orange lines show the resistance of the backward sweeps $I = -21 \rightarrow 21$ mA; the corresponding PSDs are not shown. AP, D, and DP denote Anti-Parallel, single Droplet, and Droplet Pair, respectively. Kinks at around 0 mA is an experimental artifact, which originates from non-zero voltage offset in voltmeter.

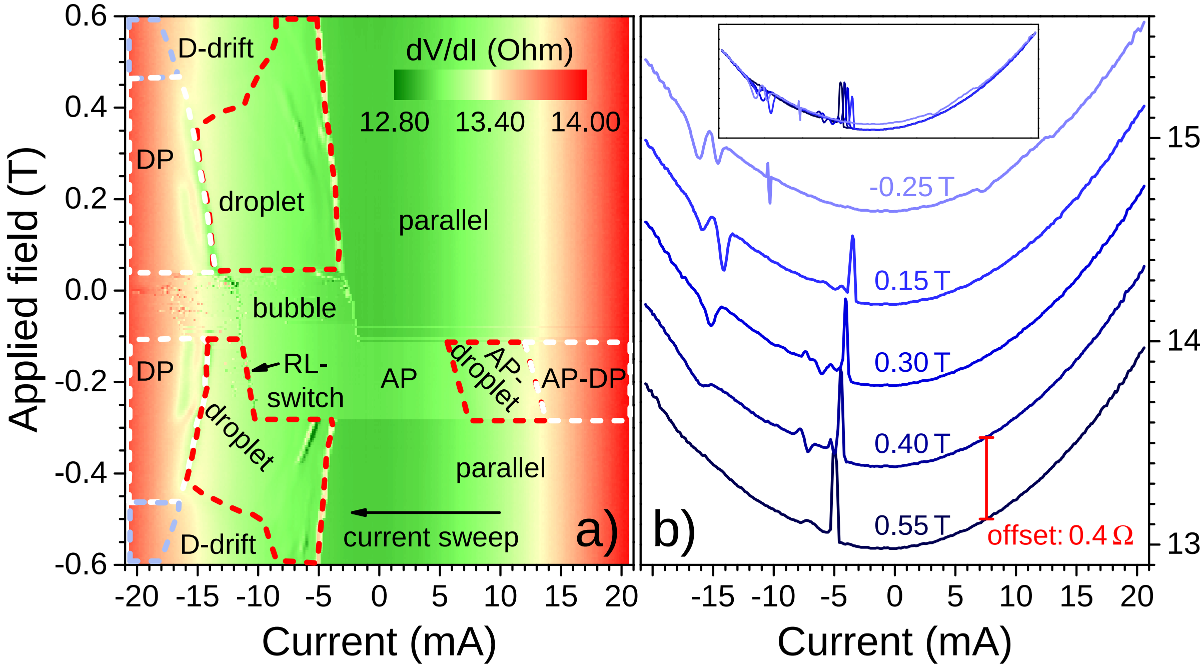

Figure S5: **Supplementary Fig. 5.** Same data as Fig. 3a in the main text, but without background correction of the differential resistance. **a** STNO differential resistance (dV/dI) as a function of applied current and field. The red lines mark regions of stable single droplets, while areas with droplet pairs (DP) are indicated by white lines. The light blue lines denote sectors where it is hard to distinguish single droplet drift (D-drift) and droplet pair dynamics. Droplets in the anti-parallel (AP) state are found at positive currents. The plain AP state transforms into a droplet state at negative currents when the reference layer (RL) magnetization switches direction. **b** The differential resistance at five representative fields. The data is offset by 0.4 Ω for clarity. The inset shows the data without offset.

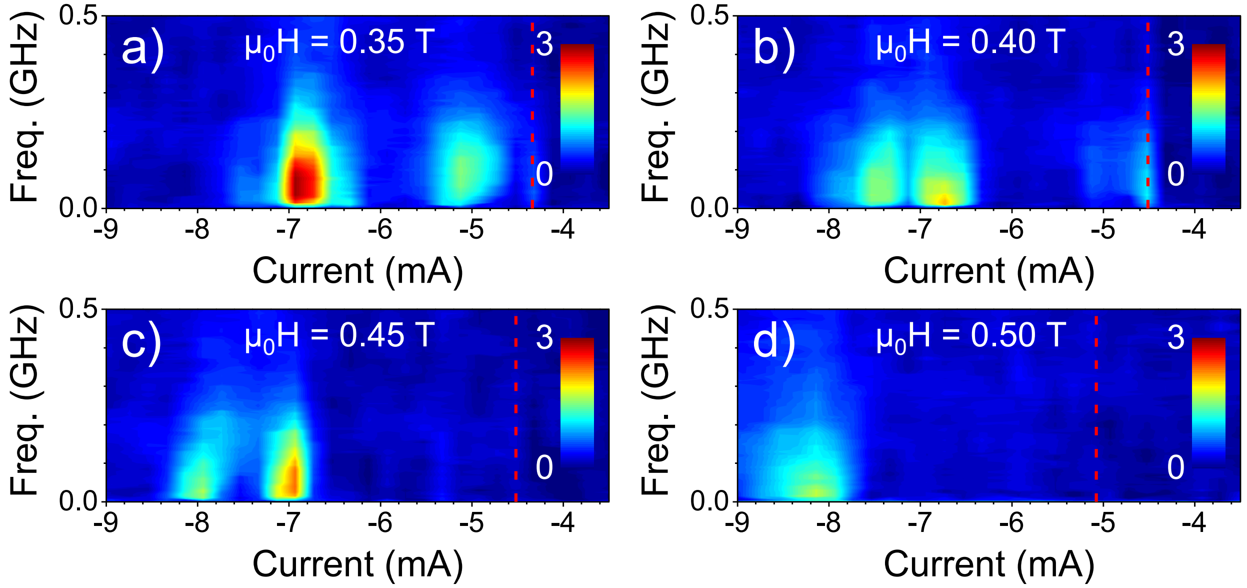

Figure S6: **Supplementary Fig. 6.** Magnification of insets in Fig. 4 in the main text. **a–d** PSD (color map) of current sweeps at four different fields: **a** $\mu_0 H = 0.35$ T, **b** $\mu_0 H = 0.40$ T, **c** $\mu_0 H = 0.45$ T, and **d** $\mu_0 H = 0.50$ T. The red dashed lines mark the nucleation of a single droplet.

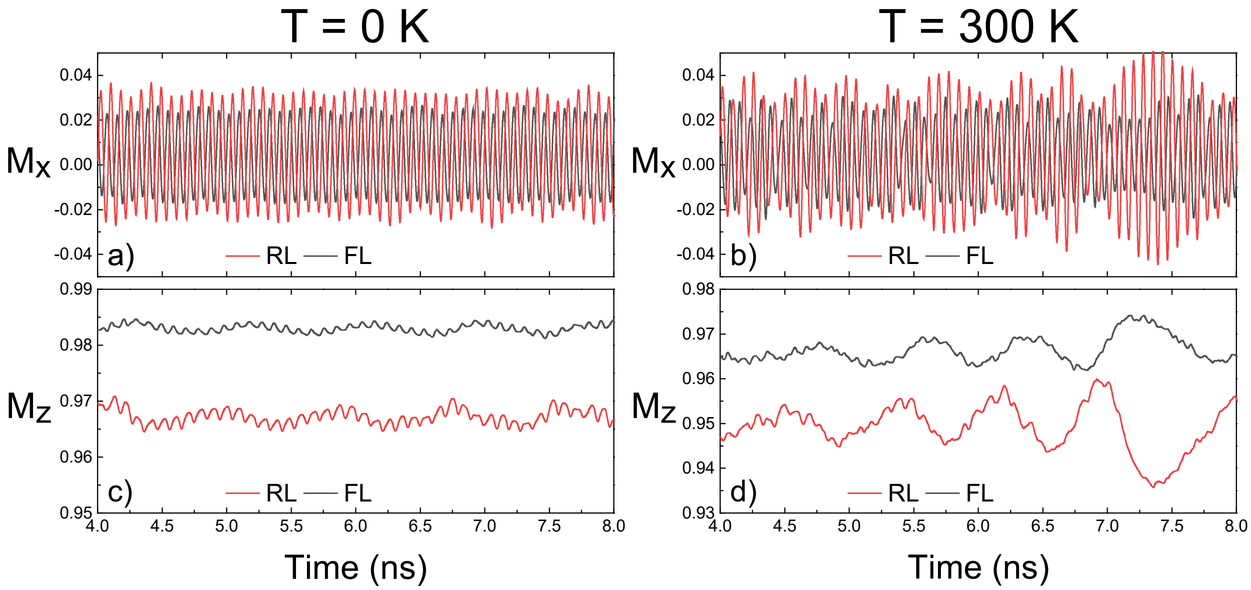

Figure S7: **Supplementary Fig. 7.** Simulation results. The **a–b** M_x-, and **c–d** M_z-components of the magnetization as function of time, at two different temperatures: **a & c** $T = 0$ K, and **b & d** $T = 300$ K. The applied field and current is $\mu_0 H = 0.5$ T and $I = -12$ mA, respectively.

Supplementary Note 1. *On the correspondence between data and simulations.*
The droplet pair nucleation current is larger in the experiments than in the simulations. We don't expect the simulations to quantitatively reproduce the experimental data. The nucleation current has a complex dependence on a variety of parameters, such as the magnetic properties, the damping, the current polarization efficiency, the current distribution, temperature, etc. It is usually possible to explore this parameter space to get a better fit to experiments, but in our case each simulation takes about a week of computing time, making exploration impossible in practice (or at least extremely time consuming). Therefore, we have reused values from former successful single droplet simulations, added reasonable estimates for the reference layer and focused on the general behavior.

In addition, the current distribution is simplified to a cylindrical flow, neglecting any in-plane current components and associated torques. Diffusive spin currents originating from the highly inhomogeneous magnetization of the polarizing layer are also omitted in the model, which only accounts for the spin polarization produced locally underneath the NC. Nonetheless, we expect that our uncomplicated model still captures the essentials of droplet pair dynamics, and that a more elaborate model would not show significantly different results.

Supplementary Note 2. *On the potential effects of diffusive spin transport.*
The droplet pair constitutes a highly inhomogeneous magnetic state, which could generate considerable diffusive spin currents due to the large gradients in spin accumulation. This kind of effects are not included in the simulations. We have calculated estimated values of the diffusive currents to compare their relative impact to the spin current carried by the applied electrical current. The result shows that the diffusive currents are two orders of magnitude lower than the accounted-for contributions, which implies that our simulations capture the most relevant torques. Details of the estimation is found below.

An applied charge current (I) flowing through a nanocontact with radius $r_\mathrm{c} = 30$ nm, generates a spin current $j_{\mathrm{s,\,nc}} = PI/\left(\pi r_\mathrm{c}^2\right) = 1.4 \cdot 10^{12}$ A/m, if the spin polarization is $P = 0.4$.

Diffusive spin currents may arise from precessing magnetic moments, an effect known as spin

pumping [1, 2]. In this case the spin current is given by:

$$\mathbf{j}_{\text{sp}} = \frac{e}{2\pi} g_{\downarrow\uparrow} (\mathbf{m} \times \dot{\mathbf{m}}) \quad \rightarrow \quad e g_{\downarrow\uparrow} f = 2.1 \cdot 10^{10} \text{ A/m}^2, \tag{1}$$

where e is the elementary charge ($1.6 \cdot 10^{-19}$ C), $\mathbf{m}$ is the normalized magnetization vector ($|\mathbf{m}| = 1$), $g_{\downarrow\uparrow}$ is the spin mixing conductance ($\simeq 10^{-19}$ m^{-2} [3]) of the [Co/Ni]–Cu- and [Co/Pd]–Cu-interfaces, and $f = 13$ GHz is the droplet frequency. Thus, this diffusive flux of angular momentum is significantly lower than that provided by the electrical current.

Moreover, high gradients of the magnetization, which are inherent to the droplet spin texture, also result in local charge currents that need to be considered. This three-dimensional current is driven by a spin "motive" force, and can be calculated using [1, 4]:

$$j_i = \frac{\hbar P \sigma}{2e} \left[\mathbf{m} \cdot (\partial_i \mathbf{m} \times \dot{\mathbf{m}}) + \beta \dot{\mathbf{m}} \cdot \partial_i \mathbf{m} \right] \tag{2}$$

where $i =$ x, y, z, $\hbar$ is the reduced Planck's constant, and β is the out-of-plane spin-torque parameter. There is no significant magnetic gradient in the direction of the film normal (z-direction), since the thickness of both magnetic layers is about one exchange length ($l_{\text{ex}} \simeq 5$ nm), and we assume free interface spins. However, the in-plane components can achieve notable values. To get an estimate, we assume that the characteristic size of the droplet gradients is l_{ex}, and that the electrical conductance is $\sigma = 4 \cdot 10^6$ S/m [5]. Since $\beta \sim 0.01$ [1], we use only the left-hand term in Eq. (2), and get:

$$j_{\text{max}} = \frac{\hbar P \sigma}{2e} \frac{2\pi f}{l_{\text{ex}}} = 5.4 \cdot 10^{10} \text{ A/m}^2, \tag{3}$$

which is two orders of magnitude lower than the current density applied through the nanocontact. Therefore, we conclude that the diffusive spin current densities are substantially lower than the electrical current spin-polarized transport, despite the high gradients of a magnetic droplet.

[1] A. Brataas, Y. Tserkovnyak, G. Bauer, and P. J. Kelly, "Spin pumping and spin transfer", in *Spin Current*, 2nd ed., Sadamichi Maekawa, Sergio O. Valenzuela, Eiji Saitoh, Takashi Kimura. Oxford, United Kingdom: OUP Oxford; 2017, pp. 93–143. [arXiv:1108.0385v3]

[2] Y. Tserkovnyak, A. Brataas, and G. E. Bauer, Spin pumping and magnetization dynamics in metallic multilayers, *Phys. Rev. B* **66**, 224403 (2002).

[3] Yi Li, Wei Cao, and W. E. Bailey, Characterization of spin relaxation anisotropy in Co using spin pumping, *Phys. Rev. B* **94**, 174439 (2016).

[4] K. Hosono, J. Shibata, H. Kohno, and Y. Nozaki, Spin torques due to diffusive spin current in magnetic texture, *Phys. Rev. B* **87**, 094404 (2013).

[5] S. Chung, et al., Direct Observation of Zhang-Li Torque Expansion of Magnetic Droplet Solitons. *Phys. Rev. Lett.* **120**, 217204 (2018). [Supplementary Materials]
